# Mitochondrial DNA and Functional Investigations into the Radiosensitivity of Four Mouse Strains

**DOI:** 10.1155/2014/850460

**Published:** 2014-02-12

**Authors:** Steven B. Zhang, David Maguire, Mei Zhang, Yeping Tian, Shanmin Yang, Amy Zhang, Katherine Casey-Sawicki, Deping Han, Jun Ma, Liangjie Yin, Yongson Guo, Xiaohui Wang, Chun Chen, Alexandra Litvinchuk, Zhenhuan Zhang, Steven Swarts, Sadasivan Vidyasagar, Lurong Zhang, Paul Okunieff

**Affiliations:** ^1^Department of Radiation Oncology, University of Florida Shands Cancer Center, 2033 Mowry Road, P.O. Box 103633, Gainesville, FL 32610, USA; ^2^Department of Immunology, Second Military Medical University, Shanghai 200433, China; ^3^First Affiliated Hospital, Fujian Medical University, Fuzhou, Fujian 350108, China; ^4^Institute of Digestive Diseases, Zhengzhou University, Henan 45001, China; ^5^Department of Cardiovascular Diseases, Hospital of Fujian Province, Fuzhou 350004, China; ^6^Department of Physiology, Shanghai University of Sport, Shanghai 100044, China; ^7^Institute of Radiobiology, National Academy of Sciences of Belarus, 220072 Gomel, Belarus

## Abstract

We investigated whether genetic radiosensitivity-related changes in mtDNA/nDNA ratios are significant to mitochondrial function and if a material effect on mtDNA content and function exists. BALB/c (radiosensitive), C57BL/6 (radioresistant), and F1 hybrid mouse strains were exposed to total body irradiation. Hepatic genomic DNA was extracted, and mitochondria were isolated. Mitochondrial oxygen consumption, ROS, and calcium-induced mitochondrial swelling were measured. Radiation influenced strain-specific survival *in vivo*. F1 hybrid survival was influenced by maternal input. Changes in mitochondrial content corresponded to survival *in vivo* among the 4 strains. Calcium-induced mitochondrial swelling was strain dependent. Isolated mitochondria from BALB/c mice were significantly more sensitive to calcium overload than mitochondria from C57BL/6 mice. Maternal input partially influenced the recovery effect of radiation on calcium-induced mitochondrial swelling in F1 hybrids; the hybrid with a radiosensitive maternal lineage exhibited a lower rate of recovery. Hybrids had a survival rate that was biased toward maternal input. mtDNA content and mitochondrial permeability transition pores (MPTP) measured in these strains before irradiation reflected a dominant input from the parent. After irradiation, the MPTP opened sooner in radiosensitive and hybrid strains, likely triggering intrinsic apoptotic pathways. These findings have important implications for translation into predictors of radiation sensitivity/resistance.

## 1. Introduction

Although much research has focused on nuclear DNA (nDNA) as a potential site for radiation damage, attention has now turned to the responses of mitochondrial DNA (mtDNA) to ionizing radiation. While mtDNA and nDNA have chemically identical gross compositions, subtle differences in their subcellular environments lead to different responses to radiation. Shortly after irradiation, many of the changes induced in DNA are spontaneously reversed. Most of the remaining alterations in nDNA are repaired by different enzyme-mediated pathways. Although a limited level of mtDNA repair does occur, most of the repair pathways do not exist in mitochondria [1]. Nuclear DNA damage is restricted by the presence of proteins (histones) that shield DNA from radiation. Conversely, the mitochondrial genome has limited (no histone) protein protection.

It has been suggested that the many mtDNA copies in each mitochondria and the large number of mitochondria in each cell are insurance against extensive disruption to cellular function, particularly respiration following irradiation [2, 3]. This phenomenon suggests the existence of “proofreading” of individual mitochondrial function that is linked to an active mechanism that removes defective mitochondria. Such a mechanism would be necessary to avoid overburdening cells with dysfunctional or nonfunctional mitochondria.

Following irradiation, plasma genomic DNA copy number increases dramatically in a dose-dependent and time-dependent manner [3–5]. Following total body irradiation (TBI), mice and rats exhibit a dose-dependent peak increase at 24 hours and then a slow decrease over several months. Long-term muscle studies have shown that mtDNA levels as compared to nDNA levels are below control values, even when corrected for the age-dependent decrease in the ratio [6]. No proven mitigation agent for this mtDNA response to radiation exists.

There is emerging evidence for mtDNA changes in the complex processes of cellular aging and neoplasia [7–9]. Mitochondrial DNA protein mutations have been identified in many types of tumors [8, 10], and a role for mutations in mitochondrial DNA has been suggested by recent studies that found a high frequency of mtDNA mutations in a variety of cancers, including colon, bladder, and lung [10, 11]. Indeed, many mitochondrial genes are well-defined tumor suppressors and are associated with signaling cascades that promote cellular death [8, 12, 13]. In addition, disturbance of mitochondrial function affects deoxyribonucleotide triphosphate (dNTP) balance, which in turn can result in genomic instability. Mitochondrial membrane potential is an indicator of mitochondrial response to external and internal stimuli and plays a key role in sensitivity or resistance to both radiation and oxidative stress.

In this study, we aimed to determine if mitochondrial DNA, which is maternally inherited, plays a role in mouse strain-specific radiosensitivity. The significance of changes in mtDNA/nDNA ratios from radiosensitive (BALB/c), radioresistant (C57BL/6), and F1 hybrid (BALB/c female × C57BL/6 male and BALB/c male × C57BL/6 female) mouse strains was examined. In addition, mitochondrial physiological levels of adenosine-5′-triphosphate (ATP), reactive oxygen species (ROS), and calcium-induced mitochondrial permeability transition pore (MPTP) swelling were measured. We found that mitochondrial copy number and the MPTP are strongly associated with mouse strain radiosensitivity and that this sensitivity is maternally inherited.

## 2. Materials and Methods

### 2.1. Ethics Statement

This study was performed in strict accordance with University of Rochester Institute Animal Protocol. Care and use procedures were approved by the Institutional Animal Care and Use Committee.

### 2.2. Animals and Treatments

Male and female 7- to 8-week-old BALB/c and C57BL/6 mice (National Cancer Institute's Mouse Repository, Frederick, MD) were housed in a microisolator colony management room at the University of Rochester (Rochester, NY) on a 12-hour light/dark cycle and fed a standard diet. Five days after arriving, the mice (except for breeding mice) were immobilized in plexiglass boxes and sham irradiated (control) or administered a single fraction of TBI using a cesium-137 gamma source at a dose rate of 1.75 Gy/minute. After irradiation, mice were monitored daily for one month. Following ICAU protocol, BALB/c females were bred with C57BL/6 males and BALB/c males were bred with C57BL/6 females to produce F1 hybrid strains. Hybrid mice were treated with the same procedures described above.

### 2.3. Collection of Tissues and Isolation of Hepatic Mitochondria

In a previous study, we tested genomic DNA from different tissues and found that the liver was sensitive to radiation. Thus, in this study mice were euthanized through decapitation for liver tissue collection. The collected tissues were either used immediately or frozen at −70°C until use. Livers were placed into ice-cold buffer *A* (250 mM sucrose, 1 mM EGTA (ethylene glycol tetraacetic acid), and 10 mM HEPES (4-(2-hydroxyethyl)-1-piperazineethanesulfonic acid) (pH 7.4)). Tissues were chopped finely, and the buffer *A* was changed 3 to 5 times during this procedure to remove the blood cells and glycogen. The chopped tissues were homogenized with a glass/teflon potter tissue grinder for up to 5 minutes. The homogenized tissues were centrifuged for 10 minutes at 500 ×g at 4°C. The supernatants were transferred into centrifugation tubes and centrifuged for 10 minutes at 12000 ×g at 4°C. Then the sediments were resuspended with buffer *B* (250 mM sucrose and 10 mM HEPES, pH 7.4) and centrifuged again for 10 minutes at 12000 ×g at 4°C. The pellets were resuspended with 0.7 mL buffer *B* and transferred into 1.5 mL microfuge tubes. The isolated mitochondria were kept on ice and used within 4 hours after the isolation procedure was completed.

### 2.4. DNA Extraction of Tissues and Isolated Mitochondria

Total genomic DNA was extracted from liver tissues. Mitochondrial DNA was isolated from the hepatic mitochondria by standard proteolytic digestion followed by phenol/chloroform/isoamyl alcohol purification. The extracted DNA was diluted in TE buffer (T_10_E_1_, pH 8.0), and its absorbance was measured in an ultraviolet spectrometer at 260 nm and 280 nm for quantification and quality assessment.

### 2.5. Protein Quantity of Isolated Mitochondria

Isolated mitochondria were diluted with cell lysis buffer (100 mM NaCl, 10 mM Tris-Cl, (pH 8.0), 25 mM EDTA (pH 8.0), and 0.5% sodium dodecyl sulfate) using a BCA (bicinchoninic acid) protein assay kit (Thermo Scientific, Rockford, IL). The sample protein concentration was calculated by reference to a protein standard curve using bovine serum albumin as the standard.

### 2.6. Mitochondrial DNA Number

The ratio of mitochondrial DNA to nuclear DNA was determined as described by Zhang et al. [3]. The 18S rRNA mouse nuclear gene target and the 12S rRNA gene target of the mouse mitochondrial genome were amplified. Quantitative polymerase chain reaction (PCR) analysis using SYBR green (AB Science, Paris, France) was performed with 50 ng isolated DNA on iQ5 Optical System Software (Bio-Rad, Laboratories, Inc., Hercules, CA). The PCR reaction was subjected to an initial denaturation at 95°C for 10 minutes, followed by a 40 amplification cycle of denaturation at 95°C for 15 seconds, annealing at 59°C for 25 seconds, and extension at 72°C for 50 seconds. The cycle number (Ct) was recorded at the point when fluorescence crossed the threshold value. The relative amount of mtDNA to nDNA was determined by comparison of 12S rRNA to 18 S rRNA Ct levels. The targets were the nDNA gene that codes for 18S ribosomal RNA and the mtDNA gene that codes for 12 S ribosomal RNA.

### 2.7. Mitochondrial Oxygen Consumption

Oxygen consumption of mitochondria isolated from mouse livers (1 mg protein) was measured with a Clark-type electrode (Hansatech Instruments Limited, Norfolk, UK). Samples were diluted with mitochondrial buffer *B* supplemented with 0.01 mM EGTA (to chelate Ca^2+^ impurities in the water), 1 mM Mg^2+^, and 5 mM potassium phosphate. After 2-3 minutes, the respiratory chain activation of the mitochondria was recorded with the addition of 5 mM succinate, followed by 0.1 mM ADP for up to 2 minutes, 1 mM ADP for up to 3 minutes, and 0.1 mM atractyloside to inhibit ADP/ATP exchange. To calculate the RCI (7), the measured maximal O_2_ consumption in the presence of 1 mM ADP was divided by the substrate-mediated O_2_ consumption (i.e., in the presence of 5 mM succinate).

### 2.8. Mitochondrial Reactive Oxygen Species

Mitochondrial ROS was measured with an Amplex Red Assay (Invitrogen Corporation, Carlsbad, CA) [14, 15]. Isolated hepatic mitochondria (1 mg) were suspended in respiratory buffer (2 mL; 120 mM KCl, 70 mM mannitol, 25 mM sucrose, and 20 mM HEPES (pH 7.4)) supplemented with 2 U type II horseradish peroxidase, 10 nM Amplex Red, and 160 U Cu/Zn-SOD (copper/zinc-superoxide dismutase) (Sigma-Aldrich, St. Louis, MO). Fluorescence was measured in a cuvette with a Shimadzu RF 5301 spectrofluorometer (Shimadzu Scientific Instruments, Columbia, MD) in a cuvette. Once the O_2_ concentration in the liquid phase reached a steady state, fluorescence was monitored for at least 3 minutes to establish the rate of ROS generation, which was then calibrated by the addition of 1 *μ*M actinomycin followed by 1 *μ*M H_2_O_2_. We did not add SOD to maximize H_2_O_2_ measurement in these incubations because the rate was the same whether or not SOD (80 U/mL of Cu/Zn-SOD) was present. This result was likely due to either the high endogenous rate of spontaneous dismutation of O_2_^∙−^ or the fact that residual SOD remained in the mitochondrial preparation. All absorbance spectra were recorded at a steady state (O_2_) using a wavelength scan between 400 and 650 nm. H_2_O_2_ generation was calibrated by constructing standard curves using known H_2_O_2_ concentrations. ROS concentrations were determined from the slope of the fluorescence change.

### 2.9. Mitochondrial Swelling

Mitochondrial swelling was induced using the method described by Petronilli et al. [16]. Isolated hepatic mitochondria (1 mg of protein) were diluted in 1 mL of the modified mitochondrial swelling buffer (120 mM KCl, 65 mM sucrose, 2 mM succinate, 5 mM Na_2_HPO_4_/NaH_2_PO_4_ (pH 7.4), and 10 mM HEPES (pH 7.2)). The change in absorbance was recorded with a Beckman DU-640 spectrophotometer (Beckman Coulter, Inc., Brea, CA) at 540 nM for 2~3 minutes to obtain a stable baseline; thereafter, mitochondrial swelling was induced, followed by the addition of either Ca^2+^ to induce mitochondrial swelling or 200 nM CSA for 5 minutes and then a subsequent addition of Ca^2+^ to induce mitochondrial swelling.

### 2.10. Statistical Analysis

Values are expressed as means ± SD. Comparisons were made using either a one-way or two-way analysis of variance followed by a Student's *t*-test. Significant differences were determined at *P* < 0.05.

## 3. Results

### 3.1. Mouse Survival Studies

**Figure 1:**
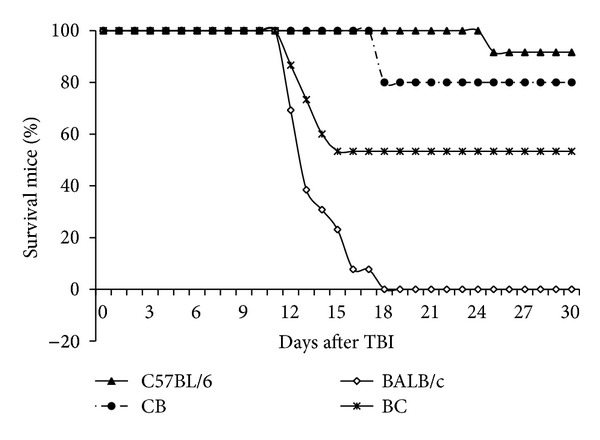
Effect of radiation on survival (LD_50/30_) in 4 mouse strains. In groups of 7~13, mice were exposed to 7 Gy TBI from a cesium-137 gamma source. Mouse survival and death rates were recorded. Significant differences existed between the C57BL/6 and BALB/c strains; survival in the F1 hybrids was significantly increased as compared to the BALB/c strain.

Figure 1 shows the radiation dose-response curves for the purebred and crossbred mice. C57BL/6 female × BALB/c male hybrids (*n* = 12) were tolerant of 7 Gy TBI with no gastrointestinal syndrome deaths (7–10 days) and rarely any bone marrow syndrome deaths (10 to 30 days). In contrast, the BALB/c female × C57BL/6 male hybrids (*n* = 15) died of bone marrow syndrome, indicating a LD_50/30_. The difference in survival rates was significant (*P* < 0.05). The earlier deaths of mice with BALB/c mothers suggest that these animals have more radiosensitive enteric and bone marrow cells. The offspring of the reverse cross (radioresistant mother with radiosensitive father) exhibited no mortality until day 18, and the ultimate survival rate was not significantly different from that of the maternal line (*P* ≈ 0.30).

### 3.2. Mitochondrial Genome Ratios

**Figure 2:**
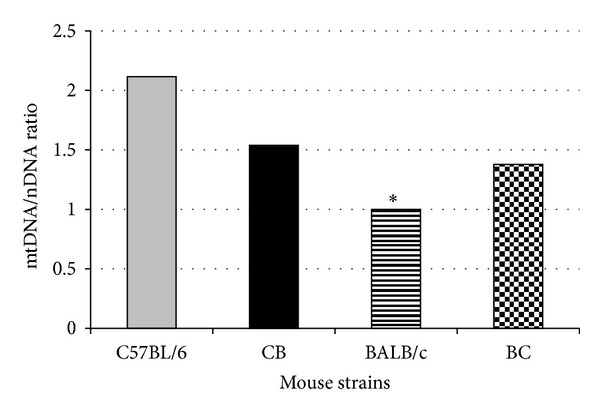
mtDNA to nDNA ratios in livers of C57BL/6, BALB/c, and F1 hybrids. A significant difference (*P* < 0.01) existed between C57BL/6 and BALB/c mice. The mtDNA to nDNA ratios of the F1 hybrids were higher than those of BALB/c mice but lower than those of C57BL/6 mice.

To identify any strain-specific differences, we estimated the ratio of mtDNA to nDNA in all F1 generation mice. As Figure 2 shows, there was a significant difference in mtDNA/nDNA ratios between the 2 parental strains; the more sensitive strain had less mtDNA per cell than the resistant strain. The 2 hybrid strains had a statistically similar ratio of mtDNA to nDNA. All strains exhibited similar responses after irradiation (data not shown).

### 3.3. Mitochondrial Oxygen Consumption

Oxygen consumption in isolated mitochondria from mouse livers was determined using standard protocol [17] and a Clark-type electrode. Oxygen consumption rates were robust, as reflective of healthy mitochondria. Mitochondrial RCI values estimated from the ratio of state III to state IV oxygen consumption were also within the range indicating robust mitochondria; there was no difference between these values for any of the strains investigated (Table 1).

### 3.4. Mitochondrial Reactive Oxygen Species

Mitochondrial ROS production was measured in the 2 purebred and 2 hybrid strains. ROS production was robust in the mitochondria from both parents and their offspring; there were no significant differences among them (Table 1).

### 3.5. Mitochondrial Swelling

**Figure 3:**
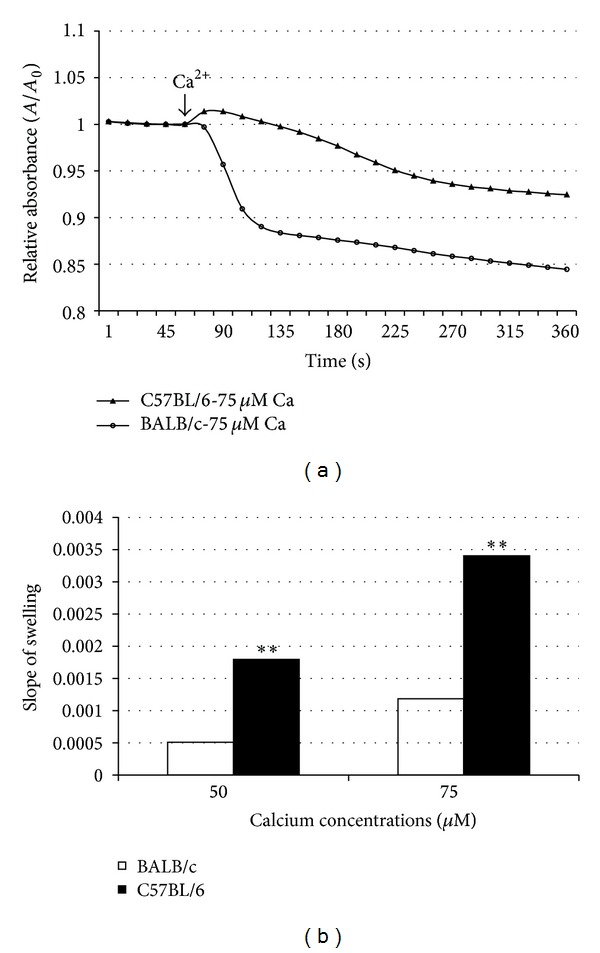
Calcium-induced hepatic mitochondrial swelling is strain dependent. Hepatic mitochondria from C57BL/6 and BALB/c mice were isolated. Two calcium concentrations (50 *μ*M and 75 *μ*M) were used to induce mitochondrial permeability pore opening. The response of mitochondria to calcium overload was recorded in 10-second intervals with a 540 nM spectrometer. Significant response differences were found.

**Figure 4:**
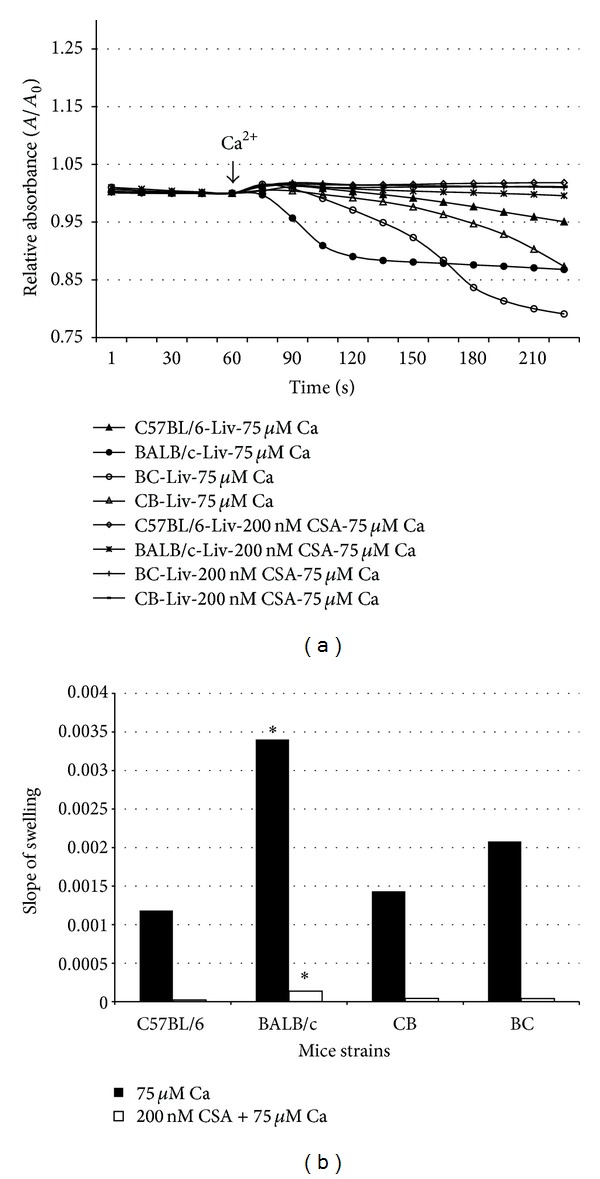
Calcium-induced hepatic mitochondrial swelling in 4 mouse strains. Hepatic mitochondria from C57BL/6, BALB/c, and C57BL/6 × BALB/c F1 hybrid mice were isolated. Calcium concentrations of 75 *μ*M with or without 200 nM CSA were used to induce mitochondrial permeability pore opening. The response of mitochondria to calcium overload was recorded in 10-second intervals with a 540 nM spectrometer. Parental background significantly influenced the response of offspring to calcium overload. CSA was found to have a significant inhibitory effect in C57BL/6 mice and the F1 hybrids.

**Figure 5:**
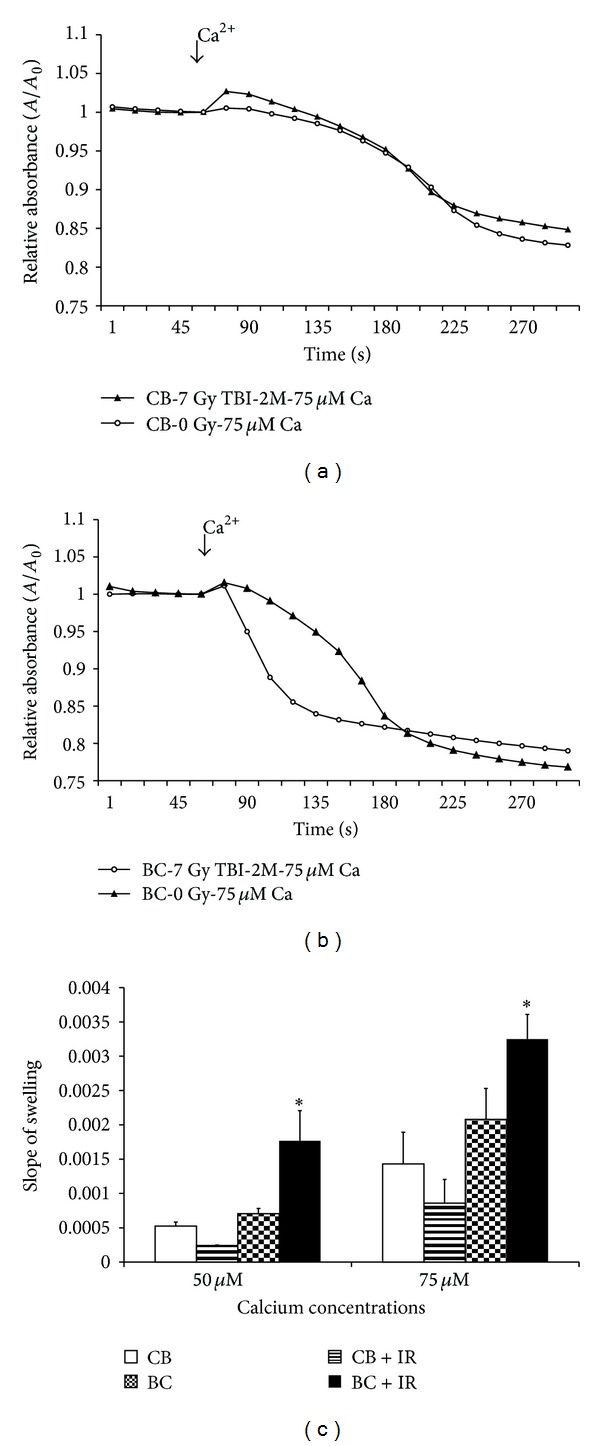
Effect of radiation on calcium-induced hepatic mitochondrial swelling. Hepatic mitochondria from C57BL/6 × BALB/c F1 hybrid mice with or without exposure to 7 Gy TBI were isolated. Calcium-induced mitochondrial permeability pore opening was tested. The response of mitochondria to calcium overload was recorded in 10-second intervals with a 540 nM spectrometer. The effect of radiation on calcium overload was found in C57BL/6 M × BALB/c F hybrid mice but not in C57BL/6 F × BALB/c M hybrid mice.

After mouse livers were excised, mitochondria were extracted as described in the Methods. Mitochondrial swelling was assessed using a standard technique, based on change of absorbance at 540 nm. In nonirradiated animals, there was a significant difference in the rate of swelling between the 2 parental strains. C57BL/6 mice exhibited a stronger resistance to calcium overload at both 50 and 75 *μ*M than the radiosensitive BALB/c mice (Figure 3). Calcium-induced swelling in hybrid strains demonstrated a moderate response to radiation (Figure 4). Maternal lineage dominated the response; C57BL/6 maternal offspring were resistant to swelling, and BALB/c maternal offspring were sensitive to swelling. Furthermore, 2 months after irradiation, the 2 hybrid strains exhibited a difference in calcium-induced mitochondrial swelling (Figure 5). Mitochondria from the hybrid with a maternal input from the Ca^++^ sensitive strain (BALB/c) was significantly more Ca^++^ sensitive than the hybrid with a paternal BALB/c background. At the calcium concentrations used, cyclosporine A (CSA, 200 nM) alleviated mitochondrial swelling in all strains (Figure 4).

## 4. Discussion

Our group and others [5, 6] have proven that (a) there is an increase in the mtDNA/nDNA ratio in a variety of mouse tissues following irradiation at moderate doses; and (b) the peak increase occurs at different times, in different tissues, in different animals, and possibly in different strains. In our DNA extraction procedures, we followed the recommended phenol chloroform extraction technique described by Kirby [18]. Swerdlow's group [19] compared extraction techniques for preparing DNA for assessment of mtDNA/nDNA ratios and concluded that the Kirby technique was the only reliable approach.

BALB/c mice are more sensitive to TBI, both with regard to acute lethality of gastrointestinal syndrome (a process that is operationally defined as death before 7 days) and bone marrow syndrome (defined as death after 7 but before 30 days). Deaths between days 7 and 10 are generally considered to be mixed deaths [20]. C57BL/6 male × BALB/c female and BALB/c male × C57BL/6 female hybrids experienced a survival rate (53% and 80%, resp.) between those of the 2 parental strains (Figure 1). This result suggests a Mendelian recessive inheritance pattern. However, the C57BL/6 maternal cross exhibited a heavy bias toward the maternal strain, which suggests some maternal dominance, such as that due to mitochondrial inheritance. The observed pattern does not fit with a purely maternal (mtDNA) inheritance in which offspring would be expected to exhibit the same level of activity as the maternal strain.

Isolated mitochondria extracted from each parental and hybrid strain were assessed by 3 independent functional assays. The respiratory control index (RCI), which is defined as the ratio of maximal respiration over substrate-induced respiration, [21] indicated the ability of isolated mitochondria to use adenosine diphosphate (ADP) for ATP generation in the presence of a substrate. There was no statistical difference between the respirations (as indicated by RCI) of the 2 parental strains (Table 1). The 2 strains exhibited similar ROS measurements; hybrid offspring expressed ROS levels similar to those of the parental strains (Table 1), again suggesting recessive inheritance. Calcium-induced mitochondrial swelling was also measured in the hepatic mitochondria of the 4 strains; the results showed differences in the strains' responses to calcium overload (Figures 3 and 4). Furthermore, 2 months after irradiation, the levels expressed in mitochondria extracted from these offspring differed significantly between the 2 strains. Mitochondria from mice with a radioresistant maternal lineage exhibited increased resistance to calcium-induced swelling. In contrast, incubation with CSA abolished any discernible difference in mitochondrial calcium-loading capacity between irradiated and nonirradiated mice of either strain.

Calcium-induced swelling is associated with an increase in mitochondrial permeability transition (i.e., the permeability of mitochondrial membranes to molecules of less than 1500 daltons) [22], which results from the opening of MPTP [23]. MPTP are protein pores formed in mitochondrial membranes under pathological conditions involving mitochondrial swelling and cell death. MPTP play an important role in some types of apoptosis. Although the exact molecular components of MPTP are ill defined, they are believed to include a number of normal mitochondrial membrane proteins, including adenine nucleotide translocase (ANT)—the complex responsible for the exchange of ATP produced in the matrix of mitochondria during oxidative phosphorylation for ADP in the cytosol.

An investigation of the reaction of human tumor cells to lethal levels of radiation showed that considerable variations existed. Radiation induced a 4977 bp deletion in mtDNA, which was found to depend on the inherent radiosensitivity of human cells [24, 25]. This research also indicated that mtDNA content appears to have high heritability, and low mtDNA content may be associated with increased risk of renal cell carcinoma. Recently, Shock et al. discovered that mitochondria play an epigenetic role [24]. The present study demonstrates that mitochondrial permeability plays a key role in energy transport and apoptosis and shows inheritable differences that are in association with radiosensitivity. The role of ANT in human tumors, such as mediating the vital exchange of cytosolic ADP and mitochondrial ATP and participating in MPTP opening via its capacity to become a lethal pore in the mitochondrial inner membrane, links to (anti)-oncogenes from the *Bax/Bcl-2* family and overexpression [25–27]. Therefore, a link between host radiosensitivity, cell mitochondrial susceptibility, and a high risk for carcinogenesis may exist.

These data indicate that irradiation *in vivo* causes an irreversible decrease in the calcium-loading capacity of mitochondria. Our future work will focus on the precise nature of the effect's mechanism following irradiation. For example, suppression of ANT content may be the key factor altering the calcium-dependent regulation of MPTP, as has been suggested for the cumulative and irreversible loss of myocardial function in patients receiving doxorubicin chemotherapy [28]. Alternatively, another factor that contributes to the formation of MPTP, such as the voltage-dependent anion channel or *Bcl2/Bcl-xl*, might be responsible for the observed resistance to calcium-induced swelling. However, the apparent association of this effect with a maternal genetic input would argue in favor of involvement by an expression product from the mitochondrial genome rather than from a nuclear-encrypted input. Thus, we plan to examine the mitochondrial genome and proteome in nonirradiated mice at various times after a range of radiation doses.

The survival of these irradiated hybrid strains was biased toward a maternal input. Mitochondrial DNA and MPTP measured before irradiation were reflective of dominant input from the parent. After irradiation, the MPTP opened sooner in the radiosensitive strain and in offspring from either mating association. It is this latter response that likely triggers intrinsic apoptosis, leading to mortality.

## Figures and Tables

**Table 1:** RCI and ROS measured in isolated hepatic mitochondria from C57BL/6 and BALB/c mice and their F1 hybrids.

Mouse strain	RCI	ROS (pMol/min/mg)
C57BL/6	3.62 ± 0.47	14.14 ± 1.36
BALB/c	3.60 ± 0.39	16.45 ± 1.95
C57BL/6 F × BALB/c M	2.87 ± 0.33	16.28 ± 2.89
BALB/c F × C57BL/6 M	3.69 ± 0.43	18.96 ± 2.09

Abbreviations: RCI: respiratory control index; ROS: reactive oxygen species.

## References

[1] Gaziev AI, Podlutskii A (2003). Low efficiency of DNA repair system in mitochondria. *Tsitologiia*.

[2] Jeng J-Y, Yeh T-S, Lee J-W, Lin S-H, Fong T-H, Hsieh R-H (2008). Maintenance of mitochondrial DNA copy number and expression are essential for preservation of mitochondrial function and cell growth. *Journal of Cellular Biochemistry*.

[3] Zhang H, Maguire D, Swarts S (2009). Replication of murine mitochondrial DNA following irradiation. *Advances in Experimental Medicine and Biology*.

[4] Zhang H, Zhang SB, Sun W (2009). B1 sequence-based real-time quantitative PCR: a sensitive method for direct measurement of mouse plasma DNA levels after gamma irradiation. *International Journal of Radiation Oncology, Biology, Physics*.

[5] Zhang L, Zhang M, Yang S (2010). A new biodosimetric method: branched DNA-based quantitative detection of B1 DNA in mouse plasma. *British Journal of Radiology*.

[6] Zhang SB, Zhang M, Cao Y (2012). Delayed effects of radiation on mitochondrial DNA in radiation-sensitive organs. *Advances in Experimental Medicine and Biology*.

[7] Wallace DC (1999). Mitochondrial diseases in man and mouse. *Science*.

[8] Copeland WC, Wachsman JT, Johnson FM, Penta JS (2002). Mitochondrial DNA alterations in cancer. *Cancer Investigation*.

[9] Eng C, Kiuru M, Fernandez MJ, Aaltonen LA (2003). A role for mitochondrial enzymes in inherited neoplasia and beyond. *Nature Reviews Cancer*.

[10] Polyak K, Li Y, Zhu H (1998). Somatic mutations of the mitochondrial genome in human colorectal tumours. *Nature Genetics*.

[11] Fliss MS, Usadel H, Caballero OL (2000). Facile detection of mitochondrial DNA mutations in tumors and bodily fluids. *Science*.

[12] Carew JS, Huang P (2002). Mitochondrial defects in cancer. *Molecular Cancer*.

[13] Wallace DC (2005). A mitochondrial paradigm of metabolic and degenerative diseases, aging, and cancer: a dawn for evolutionary medicine. *Annual Review of Genetics*.

[14] Munns SE, Lui JKC, Arthur PG (2005). Mitochondrial hydrogen peroxide production alters oxygen consumption in an oxygen-concentration-dependent manner. *Free Radical Biology and Medicine*.

[15] Starkov AA, Fiskum G (2003). Regulation of brain mitochondrial H_2_O_2_ production by membrane potential and NAD(P)H redox state. *Journal of Neurochemistry*.

[16] Petronilli V, Cola C, Massari S, Colonna R, Bernardi P (1993). Physiological effectors modify voltage sensing by the cyclosporin A- sensitive permeability transition pore of mitochondria. *The Journal of Biological Chemistry*.

[17] Frezza C, Cipolat S, Scorrano L (2007). Organelle isolation: functional mitochondria from mouse liver, muscle and cultured filroblasts. *Nature Protocols*.

[18] Kirby KS (1956). A new method for the isolation of ribonucleic acids from mammalian tissues. *The Biochemical Journal*.

[19] Guo W, Jiang L, Bhasin S, Khan SM, Swerdlow RH (2009). DNA extraction procedures meaningfully influence qPCR-based mtDNA copy number determination. *Mitochondrion*.

[20] Sharplin J, Franko AJ (1989). A quantitative histological study of strain-dependent differences in the effects of irradiation on mouse lung during the early phase. *Radiation Research*.

[21] Villani G, Attardi G (1997). In vivo control of respiration by cytochrome c oxidase in wild-type and mitochondrial DNA mutation-carrying human cells. *Proceedings of the National Academy of Sciences of the United States of America*.

[22] Menze MA, Hutchinson K, Laborde SM, Hand SC (2005). Mitochondrial permeability transition in the crustacean Artemia franciscana: absence of a calcium-regulated pore in the face of profound calcium storage. *American Journal of Physiology*.

[23] Hunter DR, Haworth RA (1979). The Ca^2+^-induced membrane transition in mitochondria. The protective mechanisms. *Archives of Biochemistry and Biophysics*.

[24] Shock LS, Thakkar PV, Peterson EJ, Moran RG, Taylor SM (2011). DNA methyltransferase 1, cytosine methylation, and cytosine hydroxymethylation in mammalian mitochondria. *Proceedings of the National Academy of Sciences of the United States of America*.

[25] Le Bras M, Borgne-Sanchez A, Touat Z (2006). Chemosensitization by knockdown of adenine nucleotide translocase-2. *Cancer Research*.

[26] Jang J-Y, Choi Y, Jeon Y-K, Kim C-W (2008). Suppression of adenine nucleotide translocase-2 by vector-based siRNA in human breast cancer cells induces apoptosis and inhibits tumor growth in vitro and in vivo. *Breast Cancer Research*.

[27] Sharaf el dein O, Mayola E, Chopineau J, Brenner C (2011). The adenine nucleotide translocase 2, a mitochondrial target for anticancer biotherapy. *Current Drug Targets*.

[28] Ong S-B, Hausenloy DJ (2010). Mitochondrial morphology and cardiovascular disease. *Cardiovascular Research*.

